# ADHD-KG: a knowledge graph of attention deficit hyperactivity disorder

**DOI:** 10.1007/s13755-023-00253-8

**Published:** 2023-11-11

**Authors:** Emmanuel Papadakis, George Baryannis, Sotiris Batsakis, Marios Adamou, Zhisheng Huang, Grigoris Antoniou

**Affiliations:** 1 School of Computing and Engineering, University of Huddersfield, Queensgate, Huddersfield, HD1 3DH West Yorkshire UK; 2 South West Yorkshire Partnership NHS Foundation Trust, Portobello Road, Wakefield, WF1 5PN West Yorkshire UK; 3 Department of Computer Science, Vrije University of Amsterdam, De Boelelaan 1081, Amsterdam, 1081 HV Netherlands

**Keywords:** Attention deficit hyperactivity disorder, Adult ADHD, Mental health, Knowledge graph, Information linking

## Abstract

**Purpose:**

Attention Deficit Hyperactivity Disorder (ADHD) is a widespread condition that affects human behaviour and can interfere with daily activities and relationships. Medication or medical information about ADHD can be found in several data sources on the Web. Such distribution of knowledge raises notable obstacles since researchers and clinicians must manually combine various sources to deeply explore aspects of ADHD. Knowledge graphs have been widely used in medical applications due to their data integration capabilities, offering rich data stores of information built from heterogeneous sources; however, general purpose knowledge graphs cannot represent knowledge in sufficient detail, thus there is an increasing interest in domain-specific knowledge graphs.

**Methods:**

In this work we propose a Knowledge Graph of ADHD. In particular, we introduce an automated procedure enabling the construction of a knowledge graph that covers knowledge from a wide range of data sources primarily focusing on adult ADHD. These include relevant literature and clinical trials, prescribed medication and their known side-effects. Data integration between these data sources is accomplished by employing a suite of information linking procedures, which aim to connect resources by relating them to common concepts found in medical thesauri.

**Results:**

The usability and appropriateness of the developed knowledge graph is evaluated through a series of use cases that illustrate its ability to enhance and accelerate information retrieval.

**Conclusion:**

The Knowledge Graph of ADHD can provide valuable assistance to researchers and clinicians in the research, training, diagnostic and treatment processes for ADHD.

## Introduction

Attention Deficit Hyperactivity Disorder (ADHD) is a widespread condition characterized by inattention, hyperactivity, and/or impulsivity that causes significant impairment across various domains. It is considered as a childhood-onset disorder that can continue into adulthood. Recent studies [1], however, suggest that adult ADHD is characterised by a broader set of symptoms and usually involves a wider range of environmental factors compared to ADHD in children. Furthermore, a significant proportion of adult ADHD patients exhibit at least one comorbid affective disorder that often leads to failure of diagnosis and are generally less responsive to commonly prescribed medication. The increased complexity of adult ADHD compared to ADHD in children and the co-occurring comorbidities raises the need for clinicians and researchers to search and combine several medical sources in order to explore the different aspects of the disorder and make appropriate and effective decisions.

Knowledge graphs have been used extensively for knowledge representation in the medical domain due to their ability to effectively integrate information from various data sources and provide explanations in processes such as diagnosis and treatment [2]. Some examples include knowledge bases for personalised medicine [3], drug interactions [4], the Universal Protein Resource (UniProt^1^) and Google’s Health Knowledge Graph. General purpose knowledge graphs, however, only provide a broad overview on a wide range of diseases or medical knowledge and cannot represent knowledge in sufficient detail necessary for capturing disease-specific particularities. Such level of detail is crucial in medical applications that focus on exploring complex structures such as adult ADHD.

Considering these limitations, we propose the construction of a knowledge graph for adult ADHD integrating a variety of resources including relevant literature, clinical trials, drug knowledge bases and general purpose medical vocabularies. ADHD-KG is an ADHD-specific graph that can transform and connect disparate sources of structured or unstructured information into a well-organised network of knowledge. ADHD-KG has been developed and tested on several use cases illustrating its suitability for supporting (a) medical queries for clinicians or researchers to explore the latest advances in ADHD literature, (b) training inquiries that aid clinicians to acquire information about ADHD and (c) clinical queries for assisting clinicians in the diagnostic and treatment procedures for patients with particular characteristics or qualities. To the best of our knowledge, ADHD-KG is the first knowledge graph in relation to ADHD. ADHD-KG version 1.0 is available at https://w3id.org/ADHD-KG.

The contribution and potential impact of ADHD-KG to relevant research communities is summarised below:

- ADHD researchers and clinicians can conduct effective automated medical question answering through ADHD-KG, reducing the time spent in manual reviews of medical literature.
- ADHD-KG supports both complex queries, integrating constraints that combine multiple information sources, as well as time-sensitive ones, associated with a particular time reference. This allows researchers to pinpoint the most relevant and up-to-date knowledge related to their area of focus.
- Artificial Intelligence researchers focusing on healthcare applications can integrate ADHD-KG with generative models to develop solutions that benefit from the inherent explainability of the former and the natural language processing capabilities of the latter.

The rest of this paper is organised as follows: background and related work is presented in Section “Background and related work”. Section “Methods” details the methodology behind developing ADHD-KG, discussing the data acquisition process, RDF conversion, information linking and knowledge graph assembly. Querying of the resulting knowledge graph for demonstrating various use cases is presented in Section “Query results using ADHD-KG” followed by evaluation and discussion in Section “Evaluation and discussion” and conclusions in Section “Conclusion”.

## Background and related work

Semantic networks representing entities of a specific domain and their properties and relations have been used in various application areas that involve complex information. Such representations are particularly useful in the medical domain where the formal machine readable semantics of knowledge graphs increase the efficiency of information retrieval processes. Furthermore when open standards such as RDF and RDFS [5] are used for the representation of information through knowledge graphs then interoperability of developed solutions and sharing of knowledge is achieved. Applications for knowledge graphs in the medical domain include, among others, information retrieval [6], medical education [7], clinical decision support [8], identification of interactions among clinical guidelines [9], personalised medicine [3] and medical data integration [4].

Massively integrated knowledge graphs, such as Google Health Knowledge Graph, LinkedLifeData^2^ or Bio2RDF,^3^ provide structured knowledge on a wide range of medical resources; however, their broader scope renders them too generic for the entities they cover. Narrowing the scope leads to knowledge graphs which emphasise on unique features of individual disorders. Kawasaki disease [10], diabetes [11], cardiovascular disease [12] and mental conditions such as depression [13] are some indicative examples of disease-specific knowledge graphs, which show great potential in assisting health experts to explore various aspects of a particular disease.

The developers of HKGB, a framework for semi-automated disease-specific knowledge graph construction [12], highlight that the construction of such knowledge graphs can be achieved due to the existence of extended semantically annotated data repositories, such as the following: MeSH medical thesaurus [14], the PubMed medical literature repository [15], the clinical trials repository [16], Side Effect Resource (SIDER) [17] and the DrugBank drug database [18]. Our work is inspired by the construction guidelines suggested blue by the developers of HKGB and includes all of the aforementioned medical resources to develop a knowledge graph for adult ADHD. In contrast to HKGB [12], which follows a semi-automated clinicians-in-the-loop approach, we focus on developing a fully automated construction process and use expert feedback in the evaluation of the fully constructed graph.

### ADHD

ADHD is a widespread condition, thus tools and resources such as the knowledge graph proposed in this work are of great importance to the wider society. ADHD is characterised by a persistent and inappropriate pattern of inattention, hyperactivity, and/or impulsivity that causes significant impairment across domains. People with ADHD also present with deficits in executive functions, behaviour and emotion regulation and motivation [19, 20]. It is estimated that within the UK, ADHD affects about 3–5% of children and 2% of adults [21], while worldwide prevalence is 2.2% in children and adolescents and 2.8% in adults.^4^

Delayed diagnosis and treatment for ADHD can be harmful to people, leading to broader mental health conditions, relationship and employment problems, criminal activities, and substance misuse. The adverse effects of untreated ADHD are well documented blue in literature, indicatively including effects on academic outcomes [22], social functioning [23], employment [24] but also life itself as ADHD can lead to increased mortality rates [25]. The fact that ADHD is both widespread and with severe effects makes clear the importance of specialised resources that will help clinicians in the diagnostic process and the treatment of the condition. This work focuses on providing such a specialised resource that facilitates the work of clinicians in improving outcomes for adults with ADHD. To the best of our knowledge this is the first such resource on ADHD.

## Methods

Our methodology followed involves combining several medical data sources to construct a knowledge graph of ADHD (henceforth ADHD-KG). This section describes each step of the construction process, including data acquisition (Section “Data acquisition”), data conversion to RDF (Section “RDF conversion”), information linking strategies (Section “Information linking”) and the final assembly of ADHD-KG (Section “ADHD-KG assembly”).

### Data acquisition

We start by defining the scope of the knowledge base. Considering the extensively large volume of literature about ADHD on the Web, this work focuses on scientific resources that specialise on adult ADHD. The proposed knowledge graph incorporates information from the data sources described next.

*Medical Subject Headings* (MeSH)^5^ is an extensive thesaurus, maintained by the National Library of Medicine (NLM), aiming to introduce consistent indexing and cataloging of biomedical literature. MeSH data is released in RDF format and is built upon the Simple Knowledge Organization System (SKOS) standard. This facilitates the creation of a hierarchical vocabulary, where terms are linked with semantic relations.

*PubMed*^6^ is a database maintained by the National Center for Biotechnology Information that specialises in biomedical and life sciences literature. Conforming to the scope of this work, we utilise the PubMed search API to retrieve scientific resources related to adult ADHD through the following query: “(adult AND (ADHD OR (attention deficit hyperactivity disorder)))”. This query yields 9537 publications in tabular format. Each resource is detailed with basic information including title, authors, publishing venue and date, abstract, PubMed ID (unique identifier in PubMed), digital object identifier and keywords.

*Clinical Trials*^7^ is an online repository of medical studies conducted on a variety of diseases or conditions, maintained by NLM, as with MeSH. Using the same keywords as above, we retrieve clinical trials that are thematically relevant to adult ADHD. Querying the database yields 660 records of medical studies formatted as XML. Each study is described with numerous fields ranging from general data, such as identifier, description, summary and keywords, to study-specific information including disease being studied, associated interventions and drugs being used or external biomedical sources associated with the study.

*Side Effect Resource* [17] (SIDER): is a collection of recorded adverse effects caused by marketed medicine. ADHD-KG includes a complete image of SIDER dataset, which is available as a collection of tabular data containing 1430 drug entries. Each entry contains naming information about a drug, its adverse reactions, their recorded frequency and side effect classifications.

*DrugBank* [18] is a database that combines cheminformatics and bioinformatics resources to compile a detailed collection of drug data with comprehensive drug target information. The available data contains 14594 drug entries released as a large XML file structured according to the DrugBank built-in schema. Each drug is detailed with an extensive set of properties covering various characteristics, such as designation, description, classification, drug interactions or pharmacological, chemical and pharmaceutical information.

### RDF conversion

**Fig. 1:**
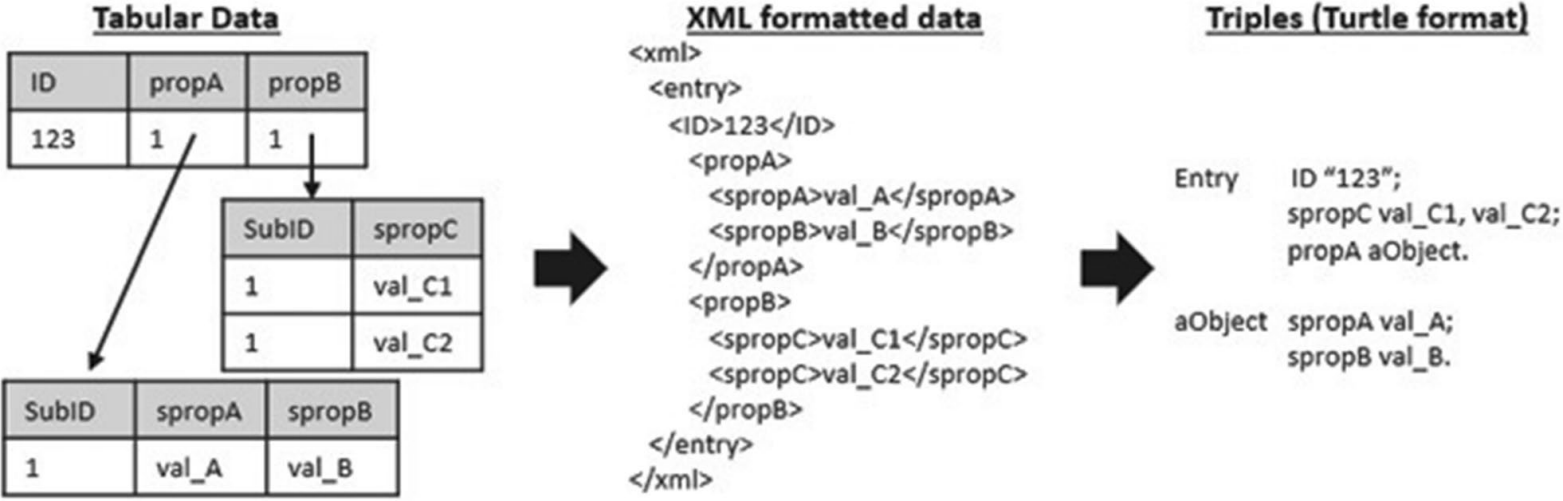
Conversion of data sources to RDF

Most of the knowledge sources described in Section “Data acquisition” make their data available either in XML format or as a collection of tabular data in comma-separated value format (henceforth CSV), where each data entry conforms to a custom schema specified by the data provider. In order to enable data integration, data discrepancies both in format and schema must be resolved. A generalised data transformation algorithm is proposed to convert data that are stored in XML or CSV formats, into RDF triples. Figure 1 illustrates this transformation process, which begins with the conversion of any tabular data into XML. A table is converted into a collection of XML entries, where each row represents an entry and columns are converted to XML tags. If a value of a column refers to external resources, then the XML tag is treated as nested and is populated by recursively decoding the contents of the referred table using the aforementioned steps. The XML formatted data are converted into triples by assuming that each XML entry is a resource and the comprised tags depict its properties. The type of property and its value depend on the contents of each tag. Particularly, ordinary tags represent data properties assigned to alphanumeric values; nested tags, on the other hand, refer to object properties with auxiliary objects as value, which are recursively populated with properties using reification.

### Information linking

**Fig. 2:**
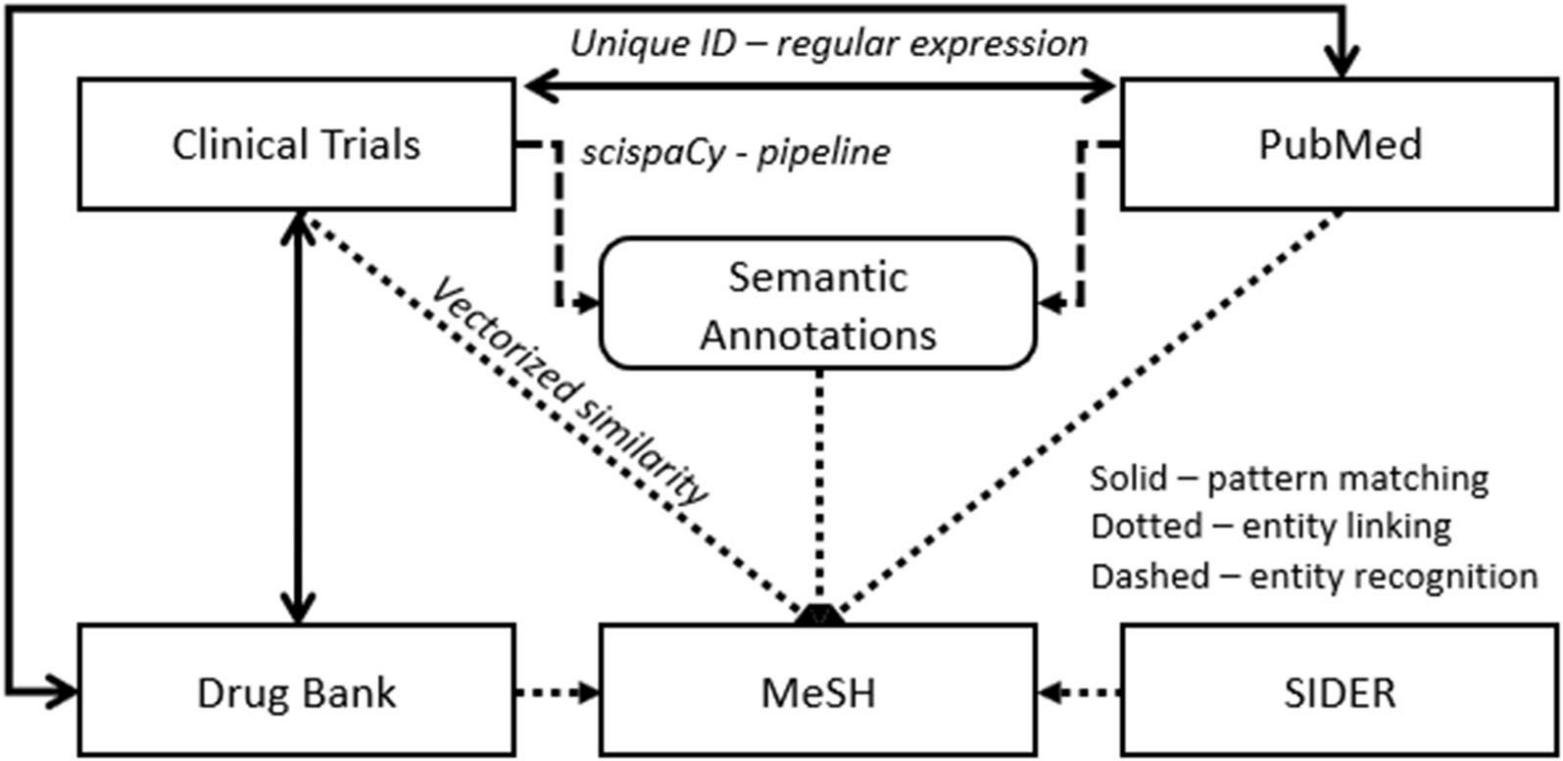
Design of the ADHD Knowledge Graph

ADHD-KG integrates five heterogeneous data sources; although each dataset specialises in unique biomedical information, semantic links between resources enable connections between different data sources. We identify two types of links: direct information linking occurs when a data source contains explicit references to resources defined in an external dataset; indirect information linking, on the other hand, is realised when disparate data sources contain resources that refer to a common medical concept. Regardless of the type of linkage, connections between data sources are expressed as custom RDF statements that associate the related resources. Figure 2 illustrates the information linking process.

#### Direct information linking

For indexing purposes, entities within knowledge bases are assigned to unique identifiers, which can also be used by external sources to refer to the entity in question. For instance, a report of a clinical trial refers to the PubMed ID of a publication to facilitate direct retrieval from the PubMed repository. These direct references introduce direct connections between data sources, which can be mined via regular expressions against the format of the unique identifiers.

Keywords are often assigned to scientific resources and serve as topic descriptors. Aligning keywords with universal vocabularies, such as MeSH, allows the association of resources with the most important concepts found in them. Detecting resources that refer to the same concept reveals a direct semantic relation, which further supports data integration. Matching keywords with concepts is accomplished through the approximate nearest neighbours search over MeSH Terms [26]. Specifically, MeSH entities are encoded as vectors using the frequency and inverse document frequency (TF-IDF) of character 3-grams and then used to create a non-metric index [27]. Employing cosine similarity, the index can retrieve the *k* most relevant MeSH concepts given a keyword and a similarity threshold (with 90% determined to be the optimal value through tuning).

#### Indirect information linking

A significant part of the information found in the data sources described in Section “Data acquisition” exists as raw text, such as titles, abstracts or simple text-based properties. Textual descriptions contain references to medical concepts, which reveal indirect associations between resources. For instance, clinical trials that mention the phrase “drug tolerance” in their title unveil a semantic correlation between them. Extracting concepts from text [28] is achieved through semantic annotations, that is, identifying meaningful terms and linking them to MeSH concepts. Among the plethora of term-based concept identifiers [29, 30], we selected scispaCy [31] for this task due to its robust and lightweight implementation, as well as the ability to resolve medical abbreviations and provide entity linking to MeSH concepts directly.

The linking procedures mentioned so far capture conceptual associations between entities by systematically linking MeSH concepts to resources. There are cases, however, where relations might exist at a finer level, such as string patterns which are not necessary linked to medical concepts. In these cases, we employed semantic queries combined with regular expressions to enable the discovery of equivocal links during query processing time. These links are generated dynamically at a cost of latency which is justified, as the process facilitates the detection of indirect associations among sources that rely explicitly on free text.

### ADHD-KG assembly

ADHD-KG is constructed through a four-step procedure: (a) data collection, (b) RDF conversion, (c) information linking and (d) graph assembly. Methodologies for steps (a) to (c) are covered in the previous subsections, while this subsection focuses on the final step which assembles all collected knowledge into a graph, shaping it according to thematic groups that reflect the design shown in Fig. 2.

**Fig. 3:**
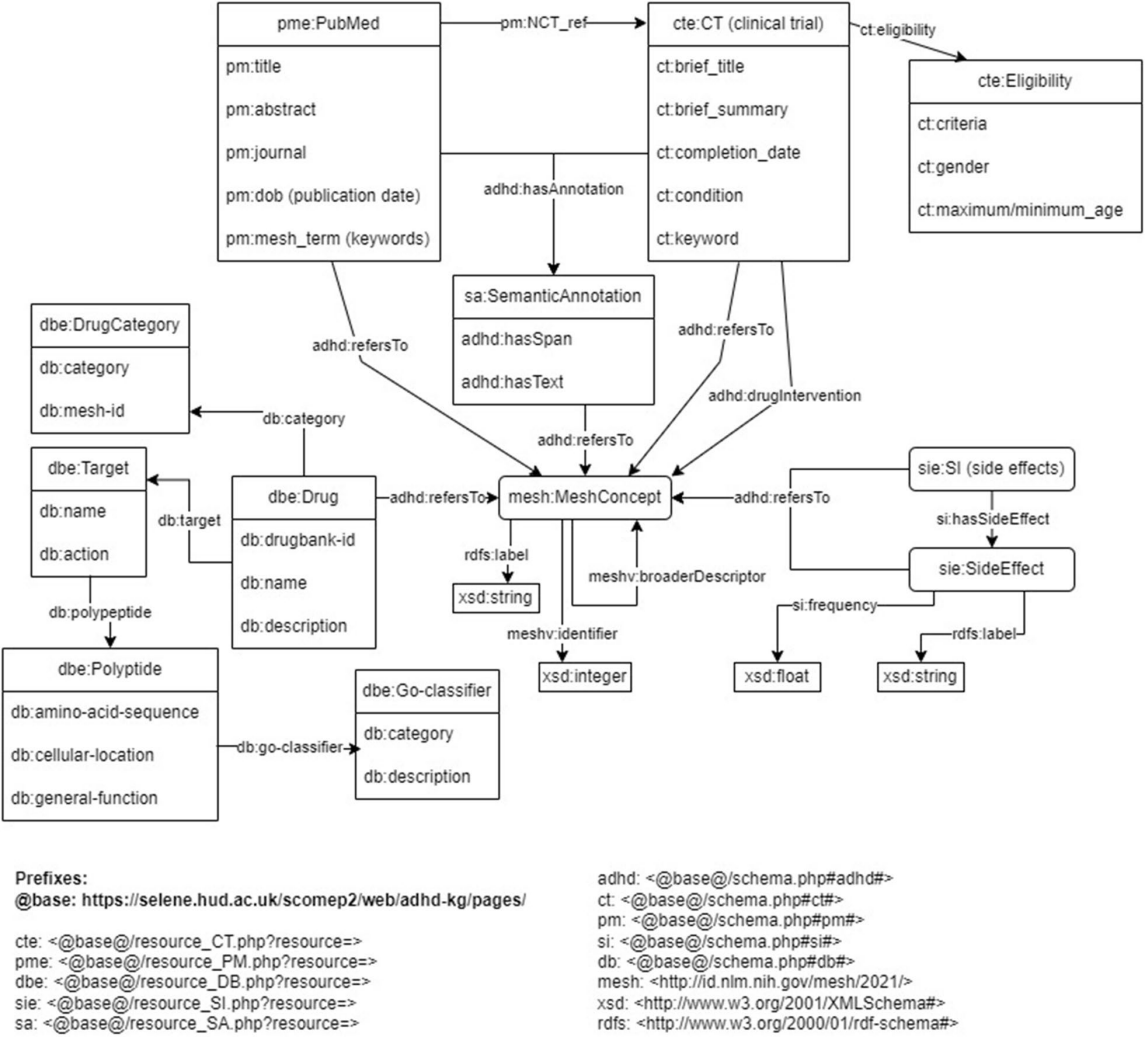
A summarised version of the ADHD-KG schema

ADHD-KG comprises six named graphs; four graphs (denoted as data graphs) store information about PubMed, Clinical Trials, DrugBank and SIDER, extended with linking statements generated during step (c). The other two graphs result from the linking procedure. The connection graph is a copy of MeSH vocabulary, which anchors the direct links from data graphs to medical concepts. Finally, the annotation graph contains semantic annotations that associate textual information from the data graphs PubMed and Clinical Trials with the connection graph. Figure 2 provides a high level schema for ADHD-KG: for schemata of individual data graphs please refer to the corresponding data resources (PubMed [15], Clinical Trials, DrugBank [18] and SIDER [17]). For the sake of convenience, Fig. 3 illustrates a summarised schema that highlights the most important concepts and relations found within the ADHD-KG. It is worth noting that each data source includes temporal references which enable ADHD-KG to answer time-sensitive queries.

## Query results using ADHD-KG

ADHD-KG is available for convenient access through a web portal,^8^ which allows users to execute semantic search and answer complex queries. In particular, this website includes information about the construction pipeline of the ADHD-KG, its schema and contents and provides functionality for resource look-up and semantic search through a SPARQL endpoint.

This section exemplifies applications of the ADHD-KG by answering complex queries through semantic search. The demonstration includes several use cases of common and prevailing ADHD-related questions, which are analysed and encoded into SPARQL queries answerable through the ADHD-KG. Due to space limitations, we provide a visualisation of the query answer instead of the query itself, where the query is too long. The graph was imported and queried in the GraphDB^9^ platform, which is a knowledge management system specialised in storing and querying RDF data.

### Keyword-based vs semantic search

**Fig. 4:**
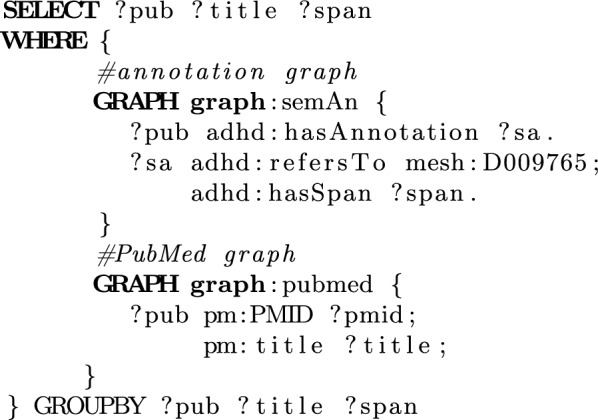
SPARQL query showing semantic search capabilities of ADHD-KG

Searching publications using keywords is limited to text similarity; semantic annotations, on the other hand, support linking between publications with medical concepts through terms that are mentioned with variable spelling, abbreviations or even synonyms. Consider the case where a researcher wants to retrieve publications about ADHD combined with obesity. This question is expressed as a SPARQL query (shown in Fig. 4), where it is examined whether a publication contains semantically annotated text that refers to the MeSH concept “D009765” (obesity). Examining the results, we find that publication “17938639” (Maternal adiposity prior to pregnancy is associated with ADHD symptoms in offspring: evidence from three prospective pregnancy cohorts) is related to obesity, even if the authors use the term “adiposity” instead.

### Information linking: drug interventions

**Fig. 5:**
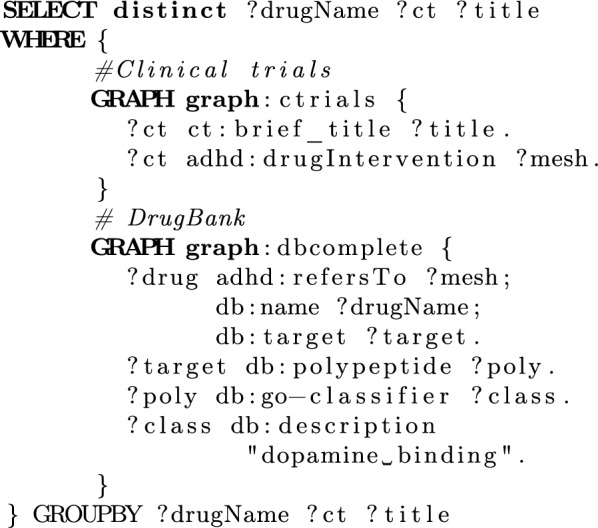
SPARQL query showing information linking capabilities of ADHD-KG, related to drug interventions

Consider a researcher who is interested in clinical trials about ADHD that conduct interventions on drugs, which target dopamine binding. Answering this question requires two tasks: first, search the Clinical Trials database to retrieve resources that conduct interventions, along with the studied drugs; then a second query must filter the retrieved drugs, through DrugBank, to determine that the final result includes only drugs with the desired action. Information linking statements within ADHD-KG allows merging these two steps into a unified query, shown in Fig. 5, which yields (indicatively) that clinical trial “NCT00150579” (“Efficacy and Safety of SPD465 in Adults with ADHD”) conducts an intervention that uses the stimulant amphetamine.

### Hierarchy of medical concepts

**Fig. 6:**
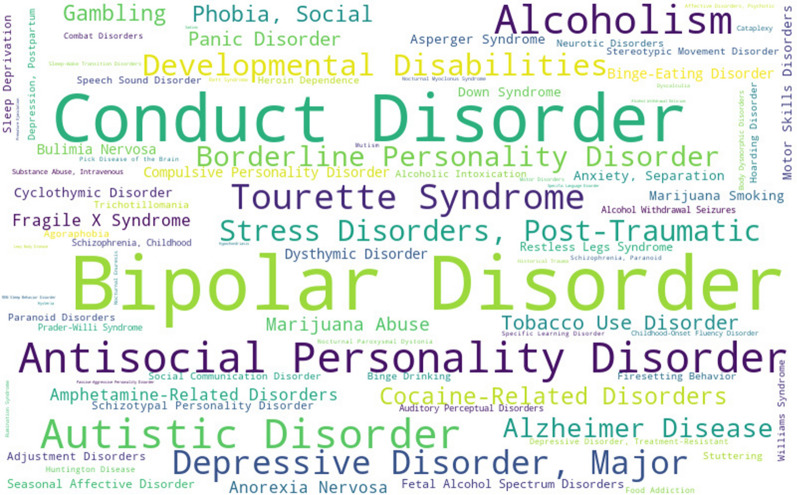
Comorbidities usually studied with adult ADHD

Diagnosis of ADHD in adults is a challenging task compared to the case of children and adolescents. It is common to diagnose adults with ADHD with more than one psychiatric disorder [32], which tends to mask or distort the underlying ADHD symptoms. A psychiatrist facing a similar situation is interested in retrieving the most frequent comorbid mental disorders found in ADHD literature. ADHD-KG can answer this complex question by leveraging data integration. A query initially scans the hierarchy of medical concept provided by MeSH to retrieve a list of mental disorders. The retrieved pool of psychopathologies is then used to select any ADHD-based publication that contains explicit (keywords) or implicit (semantic annotations) links to these comorbid disorders. The results of this query are depicted as a word cloud in Fig. 6.

### Information linking: side effects

**Fig. 7:**
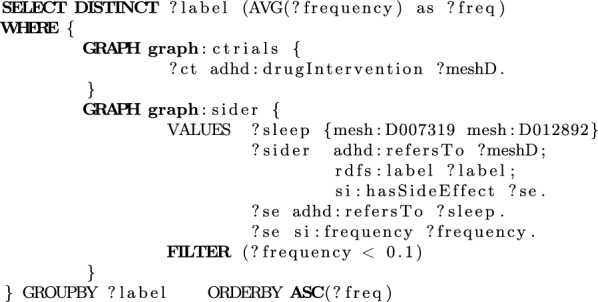
SPARQL query showing information linking capabilities of ADHD-KG, related to side effects

Consider a clinical scenario where a patient prescribed with methylphenidate complains about insomnia. In light of this information, the assigned psychiatrist is requested to find an alternative to the current medication, which has milder effects on sleep. The doctor needs to retrieve drugs that have been studied in clinical trials and filter those that have reduced sleep related side effects. This question is easily addressed via semantic search using the query illustrated below. Initially, drugs used in drug interventions are selected from the Clinical Trials graph. The retrieved drugs are joined with the SIDER graph to examine whether they have recorded side effects related to “Sleep Initiation and Maintenance Disorders” or “Sleep Deprivation”. The search concludes with the selection of drugs with a relatively low frequency of the aforementioned side effects. The corresponding query is shown in Fig. 7. Indicative results include Naltrexone, which causes insomnia with frequency 0.015%.

### Online information linking

**Fig. 8:**
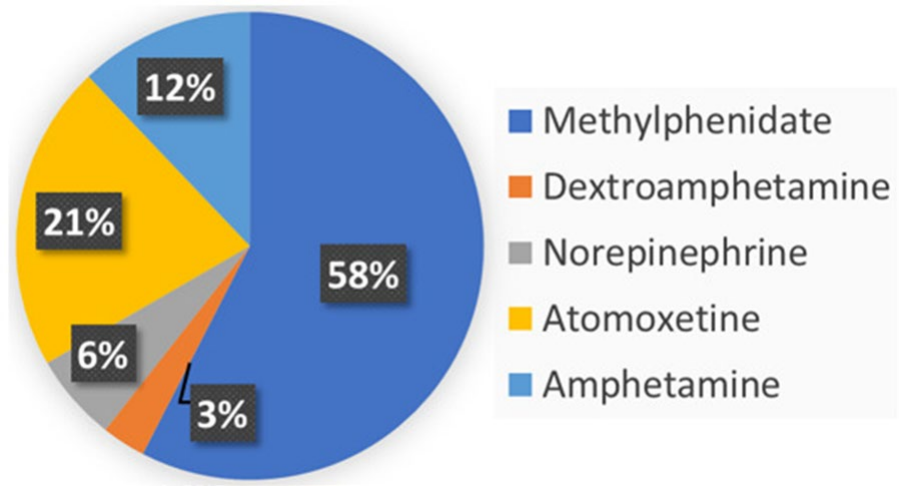
Stimulants studied for improving emotional dysregulation

Emotional dysregulation is a common complaint of people with adult ADHD that is expressed with poor management of exaggerated emotional responses. Stimulants are medicines prescribed first line to control symptoms of ADHD and some have shown a positive impact on improving emotional dysregulation. Consider the case of generating a report that summarises the frequency of such stimulants being tested in ADHD literature. This is a complex question that requires manual inspection of research work for references to emotional dysregulation and stimulants. We can express it as a query that the ADHD-KG can answer. Since emotional dysregulation is not a concept in MeSH, this query applies online information linking. In particular, semantic search in the form of regular expressions is used to retrieve publications or clinical trials that contain phrases related to “mood, emotional or behavioural dysregulation”. Results are then filtered so that only those that contain references to drugs categorised as stimulants are being selected. Figure 8 illustrates the results of this query, with “Melthyphenidate” being the most tested stimulant for improving emotional dysregulation.

## Evaluation and discussion

This section begins with a performance evaluation, focusing on graph size and average execution time. We then discuss outcomes of a preliminary validation conducted in relation to the information linking procedures and query results. Finally, we discuss potential use cases of the proposed knowledge graph, including maintenance considerations, as well as the impact of ADHD-KG to ADHD researchers and practitioners, and researchers working on applications of AI in healthcare.

### Performance

An overview of the distribution of triples contained in the ADHD-KG is shown in Table 1. Triples are organised in named graphs in order to introduce a logical structure over data without compromising interoperability, while at the same time promote a smoother and more intuitive query composition process. ADHD-KG contains 43 M triples, 6% of which corresponds to ADHD specific information, which is at a similar scale compared to other disease-specific graphs [10, 13]. It is worth noting that the graph intentionally includes complete copies of MeSH, DrugBank and SIDER data sources even for resources that are not referred to by existing ADHD publications. This strategy facilitates scalability and transferability; the graph can be extended with additional ADHD resources, such as information on ADHD in children and adolescents or adjusted to different disorders, provided that the new data sources are subjected to the processes described in Section “Methods”.

**Table 1:** Size of ADHD-KG in triples

Data source	No of Triples	% of triples
DrugBank	25 M	58.6
MeSH	15 M	35.2
SIDER	302K	0.7
Clinical trials	124K	0.3
PubMed	454K	1
Semantic annotations	1.8M	4.2

To optimise query execution time, we employ data grouping induced by named graphs, which allows for localised graph search. This can lead to a notable difference in performance because a named graph limits the total potential results of a query. For instance, searching for the drug mesh:D008774 (“Methylphenidate”) in 124K triples (Clinical trials) is more efficient compared to the full 43 M triple graph. We quantify query performance by testing an unrealistic query template that links information from every data source within ADHD-KG. Note that we exclude queries that implement online information linking, since they introduce latency that heavily depends on the complexity of the regular expression used.

The test query aims to find all drug actions (DrugBank) and side effects (SIDER) of a concept named after a known drug such as “Methylphenidate” (MeSH), which must appear as a semantic annotation (annotation graph) within publications (PubMed) or clinical trials. The average execution time recorded for the test query does not exceed 0.8 s when run on a Intel Core i7-4790 processor at 3.60GHZ with 16GB RAM. We consider such execution times as suitable for integrating ADHD-KG queries in clinical, training or research processes (as discussed in Section “Use cases”), especially taking into account that retrieving the same information using traditional means would require significant time and effort in manually searching and combining results from various data sources.

### Validation

We focused on validating two important aspects of ADHD-KG: the introduced information links and the quality of query results. For the first aspect, we employed the Unified Medical Language System (UMLS), which interconnects various biomedical vocabularies including MeSH. In particular, every pair of textual mention and medical concept is validated by online term-based queries against the UMLS Metathesaurus database.^10^ The final graph contains only results that are validated through this process.

In what concerns quality of query output, results were thoroughly examined by a team of 5 clinical experts in adult ADHD (one of which is co-author), who were involved in the planning, design and validation of ADHD-KG. These experts were contacted and asked to provide 10 questions needing answer through ADHD-KG, ranging from simple questions (e.g., what is the most effective medicine) to open questions (e.g., association of ADHD diagnosis with social stigma). The objective of the validation process was to compare answers generated by ADHD-KG to the answers expected by the experts. The criteria for evaluation were how relevant and correct the produced answers are. A detailed summary of the evaluation results is depicted in Table 2.

#### Generation of answers

Each question is transformed into a SPARQL query, which is issued against ADHD-KG and results into a number of resources. These include text summaries (e.g., publication abstracts or report summaries), semantic annotations within text or database entries such as medicine or medical concepts. Resources are used to inform the answer for the question under consideration and they result from the triple matching procedures as part of querying the ADHD-KG. Depending on the complexity of the question, aggregation is applied to provide a more direct answer. In particular, for questions that afford quantification, such as ordering entities based on some constraints, post-hoc aggregation procedures were applied to generate representative frequencies or plots.

#### Evaluation

Upon resource retrieval and generation of direct answers (when applicable), each expert was contacted separately and was asked to rate generated answers according to the criteria of relevance and correctness.

In terms of relevance, experts were presented with a random sample of 10 retrieved resources for each question, which were rated as “irrelevant” or “relevant”. Correctness was estimated by asking experts to classify the generated plots or frequencies as“valid” or “invalid”. In cases where the retrieved resources could not be aggregated, these were excluded from the estimation of the overall correctness; we denote these cases with “N/A” under correctness. The final scores of relevance per question are estimated by averaging the individual expert ratings and converting them into percentages depicting the overall agreement. For correctness, we used majority voting (e.g. the agreed classification is “valid” when at least 3 out of 5 experts classified an answer as “valid”).

#### Results

In most questions, ADHD-KG results were considered relevant and correct by experts, confirming correspondence to relevant research in ADHD. The experts did note that ADHD-KG query output sometimes included information that may be considered outdated. This is due to the fact that the queries that were executed were time insensitive, therefore an exhaustive set of results was returned. The overall relevance of the retrieved resources across all 10 questions is 77%, whereas the validity of the generated result is 85%. It is worth noting that 7 out of 10 questions were addressed with a direct answer in the form of a chart, but the remaining three (questions 6, 8 and 10) are quite elaborate to be quantified with simple aggregation. The score of 85% considers only the 7 questions were a direct answer was possible, with 6 out of 7 returning a valid result.

Elaborating on relevance results, ADHD-KG excels in addressing straightforward questions, which are based on medical concepts, for example associating medical documents with medication, side effects, proteins and so on. Relevance drops when answering questions related to textual information; this is prevalent in open questions such as patterns of ADHD onset or differences in ADHD symptoms in the presence of co-occurring conditions. Performance is expected to be affected in such scenarios, since these rely on text understanding, which is not implemented in ADHD-KG, apart from preliminary annotation of text in terms of medical concepts. Extending the annotation and connection graph (Section “ADHD-KG assembly”) with a richer medical thesauri such as SNOMED, as opposed to MeSH, can lead to major improvements in the expressiveness of the relevant queries. In order to improve ADHD-KG capabilities on addressing text-based questions, high-end natural language understanding technologies are necessary, which is an interesting idea to explore for future work. Nevertheless, question 10, which relates to the correlation of ADHD diagnosis with social stigma is excellent at showcasing the knowledge discovery potential of ADHD-KG; 70% resource relevance was achieved, despite the graph not being equipped to differentiate the textual co-occurrence of ADHD diagnosis and social stigma from an actual context-wise correlation.

**Table 2:** Evaluation of produced results in terms of correctness and resource relevance

Question	Correctness	Resource Relevance (Average)
What stimulants have side effects related to sleep?	Valid	1
What stimulants have been proven to improve emotional dysregulation?	Valid	0.9
What are the most common ADHD comorbidities?	Valid	0.86
What medicine used for ADHD targets on dopamine biding?	Valid	1
How many clinical trials involve ADHD drug interventions on participants diagnosed with obesity?	Valid	0.9
Can we predict patterns of ADHD onset, persistence and remission?	N/A	0.5
What proteins are usually associated with ADHD?	Valid	0.94
Can we prevent the onset of ADHD through early intervention?	N/A	0.3
Are ADHD presentations the same when they are accompanied by co-occurring conditions?	Invalid	0.56
Does getting an ADHD diagnosis increase or reduce social stigma?	N/A	0.7
Overall scores	0.85	0.77

It should be noted that this is a preliminary validation, thus we excluded completeness of the generated answers. We intend to follow this expert-based validation which confirmed the appropriateness of ADHD-KG in answering relevant questions, with a wider clinician-oriented validation phase as discussed in Section “Conclusion”.

### Use cases

We identify three major use cases, in which ADHD-KG has the potential to improve current practice. These relate to answering clinical, training and medical queries, as follows:

- Clinical queries include scenarios where a doctor treating a particular patient needs to investigate relevant knowledge in ADHD literature, in order to decide the best course of action. For instance, they may need to find an alternative medication that limits unwanted side effects or gain insights about a case by examining correlated clinical trials.
- Training queries focus on supporting junior clinicians in learning the basics of the ADHD domain, such as ADHD medication that impedes sleep quality or mental disorders known to hinder the ADHD diagnosis.
- Medical queries refer to queries about the latest developments in the field of ADHD, which medical experts and researchers wish to explore. These may indicatively involve applied scenarios, such as progress in using stimulants to improve emotional dysregulation, or prevailing questions, such as reviewing literature that explores whether ADHD diagnosis increases stigma.

By querying ADHD-KG instead of conducting a laborious manual search, we expect efficiency improvements in all three contexts: reduced time taken to identify appropriate treatment, faster training processes reducing the reliance of junior clinicians to senior colleagues and easier access to state of the art in adult ADHD for medical experts and researchers. In the long term, these efficiency improvements depend on the ability of ADHD-KG to account for the latest developments and debates in relevant literature, given the fact that contributions in ADHD research are quite frequent. The modular architecture of ADHD-KG allows for effective maintenance and evolution practices by enabling on-demand updates on the individual constituent graphs through the integration pipeline described in Section “Methods”. This will support frequent releases on a yearly cycle at minimum.

### Impact of ADHD-KG

In this section, we expand on the contributions of ADHD-KG to relevant academic communities, in particular researchers within the ADHD and Artificial Intelligence in healthcare fields. In addition, we provide some insights on how the proposed technology can be integrated with complementary emerging technologies using Large Language Models (LLMs).

#### ADHD research and practice

As medical practice becomes more complicated and the available literature on ADHD is increasing, experts face difficulties in retrieving the best evidence to inform their practice, research or clinical trials. This task becomes a notable challenge, when considering that medical literature is often distributed in several knowledge sources with disparate schemas (for instance, databases for medicine or clinical trial reports), raising the need for manual inspection and association of different resources. ADHD-KG simplifies information retrieval and has the potential to set the foundation for effective medical question answering. Knowledge about ADHD is integrated into a single resource, which facilitates the transition from time-consuming manual reviews of medical literature towards automated semantic search over encoded knowledge using powerful SPARQL queries.

Using the developed graph, ADHD researchers are capable of issuing queries referring to medical entities and also considering multiple sources in their search space. As a result, ADHD-KG speeds up the acquisition of medical knowledge by lifting the burden of information alignment and enabling flexible information retrieval. In particular, search with ADHD-KG goes beyond word matching, benefitting, for instance, from class hierarchies of symptoms or categories of medicine. It can also be customised using complex constraints that combine multiple information sources, e.g., retrieve every stimulant that is included in clinical trials, where participants are classified as obese. In addition to these, ADHD-KG can be useful within training processes of junior clinicians and researchers who have limited ADHD-related experience. Instead of the traditional clinical case study approach to demonstrate a learning point, senior colleagues who act as trainers can ask ADHD-KG a specific question and demonstrate the learning point from the provided answer.

Another area where ADHD-KG can have a positive impact is related to currency of knowledge. On many occasions, clinical guidelines become obsolete as soon as they are published, therefore valuable time is spent by clinicians and researchers to seek the most up-to-date knowledge. ADHD-KG supports time-sensitive information retrieval. All entries are associated with time references (e.g., publication dates, dates for changes in medicine and so on), allowing users to discern whether an entry is deprecated or contemporaneous, thus keeping track of their currency and validity. Through the frequent update cycle that is planned for ADHD-KG, we ensure that the latest information is always included.

#### Artificial intelligence in healthcare

The importance of ADHD-KG as a collection and integration of high-quality knowledge about the specific disease is topical in that it may complement perfectly recent developments in artificial intelligence and its application to healthcare. Since the publication of ChatGPT,^11^ LLMs have attracted a phenomenal attention in all sorts of domains, including healthcare and medical research [33, 34]. Despite the huge potential of this technology, there are weaknesses around the quality of its outputs, including so-called hallucinations [35], which make it less tailored to mission critical domains like healthcare. In addition, in healthcare there is often a paramount need for explainability and supporting decisions through references to high-quality sources such as medical research publications. It is in this context that ADHD-KG and similar research can play a crucial role, as such disease-specific knowledge graphs are collecting high-quality knowledge and are inherently capable of explaining the produced answers. Coupling such knowledge graphs with LLMs promises to combine the strengths of both approaches, and there is already significant research in this direction [36].

## Conclusion

In this work, we propose a procedure for building a Knowledge Graph about adult ADHD by incorporating knowledge from various relevant resources. Data integration technologies provide a well-structured network of knowledge enabling the exploration of various aspects of adult ADHD. This is the first graph for ADHD offering a rich source of information using open Semantic Web standards that can be extended for other disorders. A series of use cases, evaluated by experts, demonstrate the benefits of ADHD-KG showcasing its usability and fast response times compared to manual knowledge discovery or retrieval. ADHD-KG is designed to cover queries related to training, retrieving information about the state of the art on ADHD and assisting diagnosis and treatment.

In future work, we intend to extend the ADHD-KG with additional knowledge sources, such as proteins and genes catalogs and electronic medical records, as well as explore data fusion with additional medical thesauri, such as SNOMED. We plan to improve information linking procedures by introducing graph embeddings and through the inclusion of experts in the linking process [12]. Additionally, it is worth exploring the integration of ADHD-KG with natural language understanding components to improve question answering capabilities when relying on pure text rather than relational semantics. Finally, we intend to develop a clinician-oriented interface powered by ADHD-KG and validate its usability in actual clinical practice.

## Data Availability

ADHD-KG version 1.0 is available at https://w3id.org/ADHD-KG under a CC BY-NC-SA 4.0 licence.

## Footnotes

1 https://www.uniprot.org/

2 http://linkedlifedata.com/

3 https://bio2rdf.org/

4 https://adhd-institute.com/burden-of-adhd/epidemiology/

5 https://www.nlm.nih.gov/mesh/meshhome.html

6 https://pubmed.ncbi.nlm.nih.gov/

7 https://clinicaltrials.gov/

8 https://selene.hud.ac.uk/scomep2/web/adhd-kg

9 https://www.ontotext.com/products/graphdb/

10 https://documentation.uts.nlm.nih.gov/rest/home.html

11 https://chat.openai.com
